# Supplementary material for: Prediction of Stroke Outcome Using Natural Language Processing-Based Machine Learning of Radiology Report of Brain MRI
Source: J Pers Med. 2020 Dec 16;10(4):286. doi: 10.3390/jpm10040286 (PMC7766032; doi:10.3390/jpm10040286)
Supplement: Supplementary file 1 [file jpm-10-00286-s001.pdf]

Radiology Report of Brain Magnetic Resonance Image and Angiography

Hospital ID	OOOOOOOOO	Name	OOOOO OOOOOOOO
Referring physician	Dr. OOOOOOO	Reported by	Dr. OOOOOOO
Referring date	OOOO / OO / OO	Examined date	OOOO / OO / OO

■ **Brain MRI sequence:** Acute stroke sequence

T1/T2WI, FLAIR, GRE, DWI ADC map, TOF MRA, CE MRA (intracranial and neck)

■ **Clinical information:**

Left side weakness (2 hours ago)

■ **Findings:**

Diffusion restriction in right medial frontal cortex, right cingulate gyrus, right basal ganglia, right frontotemporal and right parietal cortex
- multifocal acute infarctions
- multifocal hemorrhagic transformation of right basal ganglia , right insular gyrus and right subinsular white matter
- No evidence of tumor, hydrocephalus, brain atrophy

MRA
- Focal severe vaso-occlusion of right proximal ACA (A1 portion)
- R/O focal vaso-occlusion of right subclavian artery (third portion)
- No evidence of aneurysm in Willis circle and cavernous ICA
- No evidence of vaso-occlusion, stenosis in Willis circle and neck carotid A

**Conclusions**	Multiple acute infarctions in right MCA territory with hemorrhagic transformation
**Recommendation**	Clinical correlation
**Confirmed date**	OOOO / OO / OO
**Confirmed Dr.**	by Dr.

Supplemental Figure S1. Example of brain MRI radiology text report.

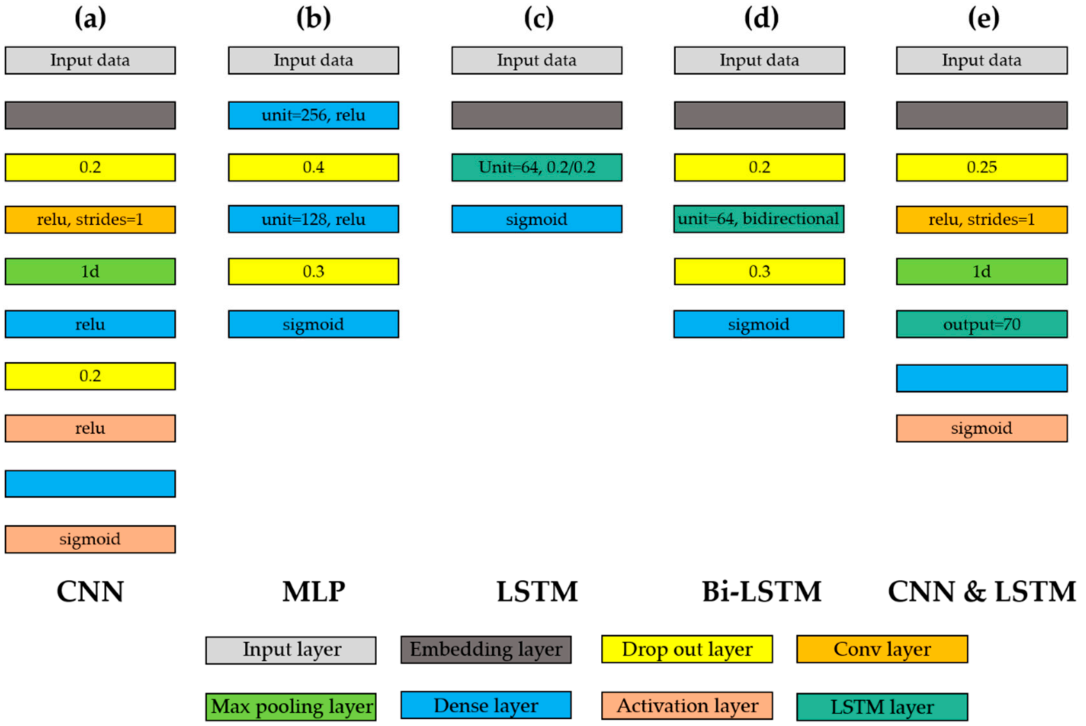

Supplemental Figure S2. Detailed architecture of the 'bag of words' model in word level approach. CNN, convolutional neural network; MLP, multilayer perceptron; LSTM, long short-term memory; Bi-LSTM, bidirectional LSTM.

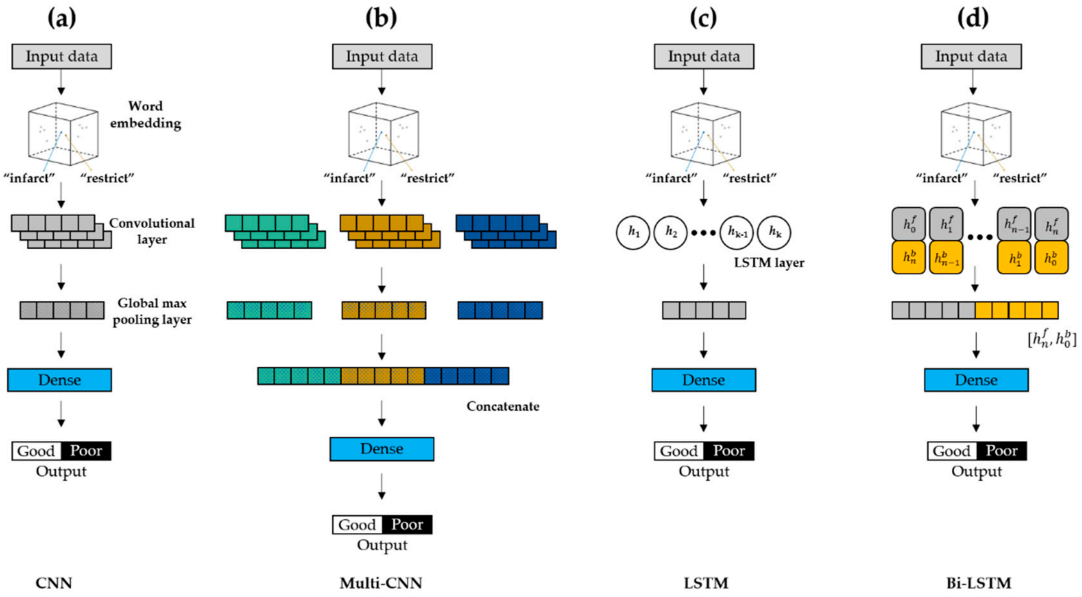

Supplemental Figure S3. Detailed architecture of the deep learning algorithms in document level approach.

CNN, convolutional neural network; Multi-CNN, multi kernel CNN; LSTM, long short-term memory; Bi-LSTM, bidirectional LSTM.

Supplemental Table S1. Most frequently used word tokens in brain MRI text dataset.

Token	Rank		
	Whole dataset	Test dataset	Training dataset
left	1st (5,236)	1st (3,720)	1st (1,516)
right	2nd (5,101)	2nd (3,632)	2nd (1,469)
infarct	3rd (4,346)	3rd (3,065)	3rd (1,281)
focal	4th (3,110)	4th (2,217)	6th (959)
diffus	5th (3,093)	5th (2,134)	4th (899)
old	6th (2,959)	6th (2,095)	7th (893)
small	7th (2,878)	7th (1,979)	5th (864)
multipl	8th (2,598)	8th (1,803)	8th (795)
matter	9th (2,299)	9th (1,625)	9th (674)
white	10th (2,233)	10th (1,574)	11th (668)
restrict	11th (2,192)	12th (1,531)	10th (659)
stenosi	12th (2,150)	11th (1,524)	12th (619)
proxim	13th (1,822)	13th (1,316)	14th (522)
mca	14th (1,770)	14th (1,273)	15th (506)
acut	15th (1,705)	15th (1,183)	13th (497)

Numbers in the parentheses represents the frequency of the word tokens that were repeated in the corresponding dataset.

Supplemental Table S2. Comparison of most frequently used tokens in training, test and whole dataset.

Order	Whole dataset		Training dataset		Test dataset	
	tokens	number	tokens	number	tokens	Number
1	left	5,236	left	3,720	left	1,516
2	right	5,101	right	3,632	right	1,469
3	infarct	4,346	infarct	3,065	infarct	1,281
4	focal	3,110	focal	2,217	diffuse	959
5	diffuse	3,093	diffuse	2,134	small	899
6	old	2,959	old	2,095	focal	893
7	small	2,878	small	1,979	old	864
8	multipl	2,598	multipl	1,803	multipl	795
9	matter	2,299	matter	1,625	matter	674
0	white	2,233	white	1,574	restrict	668
11	restrict	2,192	stenosi	1,531	white	659
12	stenosi	2,150	restrict	1,524	stenosi	619
13	proxim	1,822	proxim	1,316	acut	522
14	mca	1,770	mca	1,273	proxim	506
15	acut	1,705	acut	1,183	mca	497
16	mra	1,565	mra	1,101	mra	464
17	deep	1,496	seep	1,034	deep	462
18	ica	1,422	ica	1,001	ica	421
19	ganglia	1,394	ganglia	9,80	basal	415
20	basal	1,391	basal	976	ganglia	414

Numbers represents the frequency of the stemming word tokens that were repeated in the corresponding dataset.
